# Predictive value of extracellular volume fraction determined using enhanced computed tomography for pathological grading of clear cell renal cell carcinoma: a preliminary study

**DOI:** 10.1186/s40644-025-00866-0

**Published:** 2025-04-04

**Authors:** Jian Liu, Xunlan Zhang, Rui Lv, Xiaoyong Zhang, Rongpin Wang, Xianchun Zeng

**Affiliations:** 1 Key Laboratory of Advanced Medical Imaging and Intelligent Computing of Guizhou Province, Engineering Research Center of Text Computing & Cognitive Intelligence, State Key Laboratory of Public Big Data, College of Computer Science and Technology, Ministry of Education, Guizhou University, No. 2708, Huaxi Avenue, Guiyang, 550025 Guizhou China; 2 Department of nuclear medicine, Guizhou Provincial People’s Hospital, No. 83, Zhongshan Dong Road, Guiyang, 550002 Guizhou China; 3 Department of Radiology, International Exemplary Cooperation Base of Precision Imaging for Diagnosis and Treatment, Guizhou Provincial People’s Hospital, No. 83, Zhongshan Dong Road, Guiyang, 550002 Guizhou China

**Keywords:** Extracellular volume fraction, Clear cell renal cell carcinoma, Computed tomography

## Abstract

**Objective:**

To explore the potential of using the extracellular volume fraction (ECV), measured through enhanced computed tomography (CT), as a tool for determining the pathological grade of clear cell renal cell carcinoma (ccRCC).

**Methods:**

This retrospective study, approved by the institutional review board, included 65 patients (median age: 58.40 ± 10.84 years) who were diagnosed with ccRCC based on the nucleolar grading of the International Society of Urological Pathology (ISUP). All patients underwent preoperative abdominal enhanced CT between January 2022 and August 2024. CT features from the unenhanced, corticomedullary, nephrographic, and delayed phases were analyzed, and the extracellular volume fraction (ECV) of ccRCC was calculated by measuring CT values from regions of interest in both the unenhanced and nephrographic phases. Statistical significance was evaluated for differences in these parameters across the four ISUP grades. Additionally, diagnostic efficiency was assessed using receiver operating characteristic (ROC) curve analysis.

**Results:**

The ECV showed significant differences across the four ISUP grades of ccRCC, its potential as an important predictor of high-grade ccRCC (*P* = 0.035). The ROC curve analysis indicated that ECV exhibited the highest diagnostic efficacy for assessing the lower- and higher- pathological grade of ccRCC, with an area under the ROC curve of 0.976. The optimal diagnostic threshold for ECV was determined to be 41.64%, with a sensitivity of 91.31% and a specificity of 97.62%.

**Conclusions:**

ECV derived from enhanced CT has the potential to function as an in vivo biomarker for distinguishing between lower- and higher-grade ccRCC. This quantitative measure provides diagnostic value that extends beyond traditional qualitative CT features, offering a more precise and objective assessment of tumor grade.

**Supplementary Information:**

The online version contains supplementary material available at 10.1186/s40644-025-00866-0.

## Introduction

Renal cell carcinoma ranks as the 14th most commonly diagnosed cancer globally [1, 2]. Clear cell renal cell carcinoma (ccRCC), the most prevalent and aggressive subtype, accounts for approximately 80% of renal cell carcinoma cases [3]. The prognosis of ccRCC is closely linked to tumor differentiation, which is classified by the International Society of Urological Pathologists (ISUP) into a four-tier grading system. Tumors classified as higher grade (ISUP 3–4) typically exhibit more aggressive biological behavior and is strongly associated with poorer survival outcomes and higher recurrence rates, whereas lower grade (ISUP 1–2) is generally associated with more favorable clinical prognosis [4]. The biological characteristics and prognosis of ccRCC differ significantly by grade, necessitating distinct treatment strategies. While non-invasive/minimally invasive approaches like radiofrequency ablation, cryoablation, and active surveillance are utilized, most patients proceed to surgery immediately after diagnosis, and standardized management for these treatments remains lacking [5, 6]. Thus, accurate preoperative tumor grading is essential for guiding treatment decisions and prognosis assessment. While preoperative renal biopsy remains the “gold standard” for diagnosis, this invasive procedure carries risks such as hemorrhage, infection, and sampling errors [7]. Consequently, non-invasive preoperative methods for distinguishing between lower- and higher-grade ccRCC would be highly valuable.

There is growing interest in non-invasive imaging features and quantitative indicators for preoperative grading of ccRCC. In clinical practice, ultrasonography and computed tomography (CT) are commonly employed for evaluating ccRCC; however, qualitative assessments by radiologists are often subjective and prone to interobserver variability [8]. The extracellular volume (ECV), representing the combined volume of both extravascular and intravascular spaces, serves as an indicator of microvessel density and matrix fibrosis. As such, ECV offers a comprehensive reflection of the tumor microenvironment, capturing key aspects of tumor vascularization and extracellular matrix changes that are critical to understanding tumor biology and its progression [9, 10]. Earlier research has shown that ECV increases in various tumor types, establishing a connection between the extracellular matrix (ECM) and key processes such as tumor growth, proliferation, and invasion. This relationship underscores the role of the ECM in supporting tumor development and facilitating its aggressive behavior [11, 12].

Magnetic resonance imaging (MRI)-derived ECV fractions, based on T1 values, have proven useful in assessing tissue composition and stromal conditions. However, ECV derived from contrast-enhanced CT (CECT) has recently gained attention as a clinically feasible alternative, offering similar insights with the added advantage of widespread availability and faster imaging protocols [13–15]. CECT offers several advantages over magnetic resonance imaging, including greater accessibility, lower cost, and faster acquisition times, while also accommodating patients with contraindications to MRI. Since CECT is the preferred imaging modality for evaluating renal masses, it offers a valuable, non-invasive method for quantifying and characterizing tissue properties, including ECV. This capability makes CECT a promising tool for assessing renal tumors, facilitating detailed tissue analysis and enhancing diagnostic accuracy without the need for biopsy [16]. The application of ECV fractions derived from CECT in the evaluation of ccRCC may enhance the ability to guide personalized treatment strategies, such as focal therapy or active surveillance. However, to date, no studies have assessed the pathological grade of ccRCC using CECT-derived ECV as an index.

The objective of this study was to assess the utility of CECT-derived ECV fraction measurements in distinguishing between different pathological grades of ccRCC. Specifically, we sought to determine whether ECV values vary across pathological grades and whether CECT-derived ECV can aid in predicting the pathological grade of ccRCC.

## Methods

### Patients

**Fig. 1:**
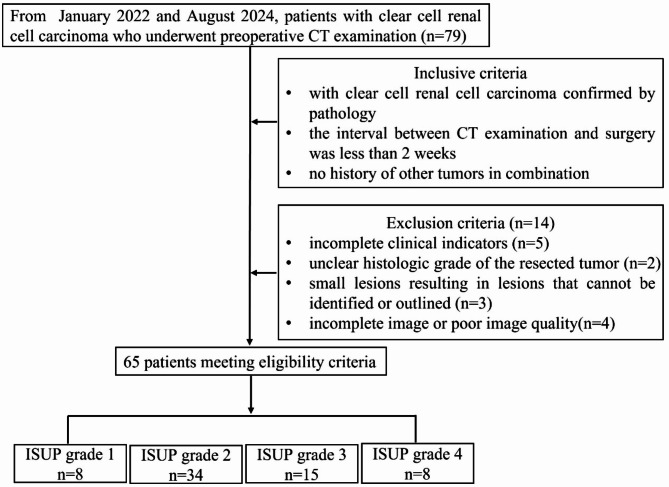
Main inclusion and exclusion criteria for the study participants

This retrospective study was approved by the institutional review board, with the requirement for informed consent waived. But all patients provided informed consent prior to undergoing CT examinations and surgical procedures. Patients enrolled between January 2022 and August 2024 were required to meet the following inclusion criteria: (1) a confirmed diagnosis of ccRCC via pathological biopsy, and (2) availability of routine CECT imaging. Exclusion criteria were as follows: (1) incomplete clinical or biochemical data, (2) absence of the lesion or poor image quality on CT scans, and (3) unclear histologic grade of the resected tumor. The study eventually included 65 patients, with a mean age of 58.40 ± 10.84 years (Fig. 1).

### CECT image acquisition

All patients underwent imaging using a third-generation dual-source CT scanner (SOMATOM Force; Siemens Healthcare, Forchheim, Germany). A standardized protocol was adhered to, with patients instructed to hold their breath during the scan and fast for 4 h prior to the procedure. The scanning parameters were as follows: a tube voltage of 120 kV, tube current of 200 mA (adjusted using the automatic tube current modulation technique, CARE Dose4D), and a slice thickness/reconstruction interval of 5 mm/5 mm. The gantry rotation time was set to 0.5 s, with a collimator width of 128 × 0.6 mm. The acquisition matrix was configured to 512 × 512. Each patient received an intravenous injection of 60–80 mL of non-ionic contrast agent (iohexol, 350 mg iodine per milliliter) into the forearm using a high-pressure syringe, with an injection flow rate of 3–4 mL/sec. The scan delay for the corticomedullary phase was determined using an automatic bolus triggering technique, with scanning initiated when the CT number of the region of interest (ROI) in the abdominal aorta reached 100 Hounsfield units (HU). All studies included four-phase scanning: after obtaining the unenhanced images, the corticomedullary phase (MP), nephrographic phase (NP), and delayed phase (DP) images were acquired at 25–30, 60–80, and 150–200 s, respectively, following the injection of contrast agent.

### Image analysis

The CECT images were evaluated using a Siemens post-processing workstation (syngo. Via, version VB40A; Siemens Healthcare, Germany). Two radiologists, with 3 and 5 years of experience respectively, independently analyzed all images, being blinded to both the clinical and pathological information. The following morphological characteristics of the tumors were assessed: long diameter, presence or absence of necrotic or cystic changes, and the presence or absence of calcification. These features were examined on both the corticomedullary and nephrographic phase images. Necrosis or cystic changes within the lesion were defined as regions with low attenuation (< 30 HU) on the nephrographic phase images. Adjustments to the window level or width were permitted to optimize the evaluation during the qualitative assessment.

**Fig. 2:**
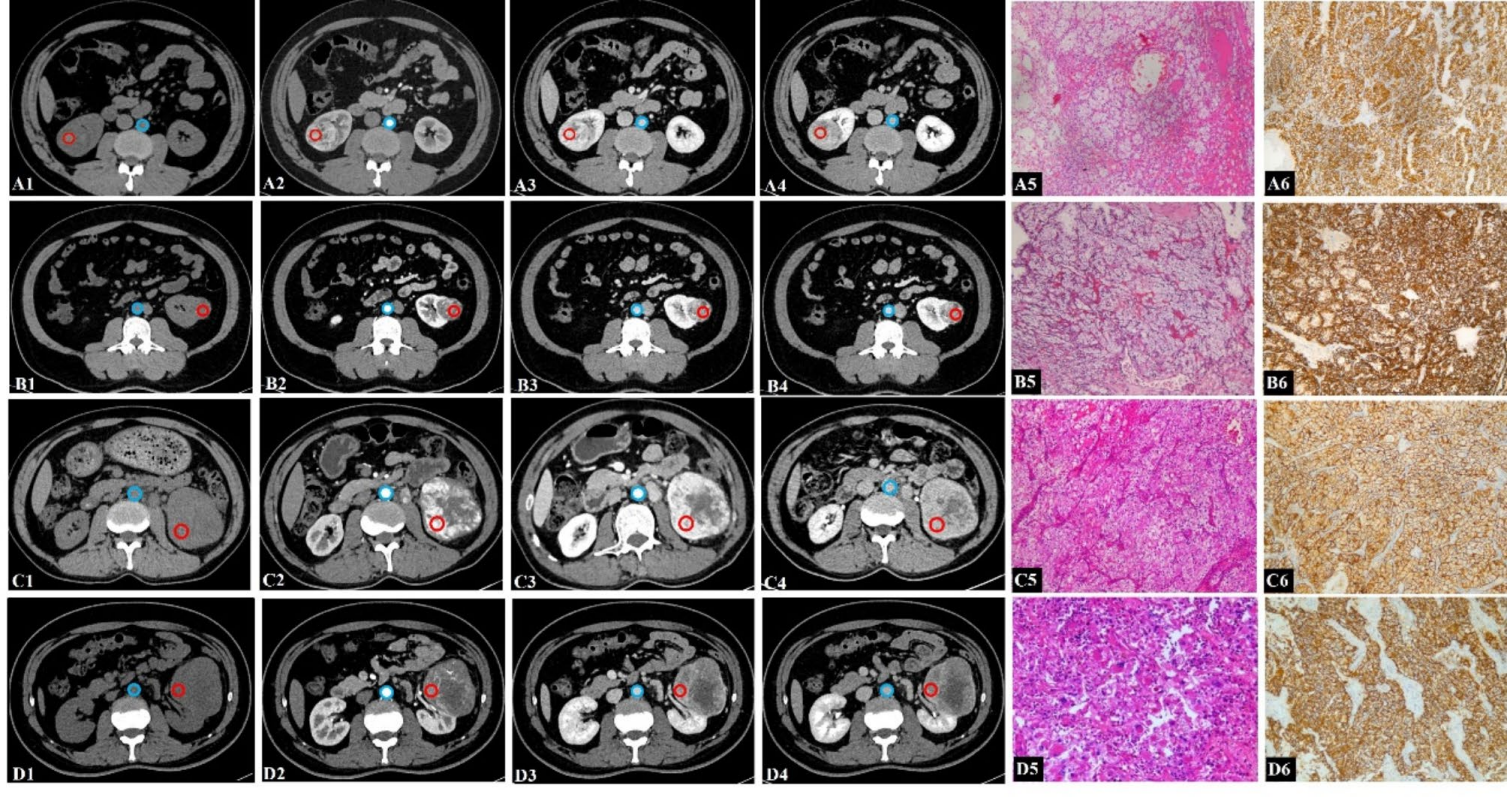
CT values were measured in the ccRCC and aorta of typical case diagram. Placement of regions of interest in the aorta (red circle) and ccRCC (blue circle) avoiding stair-step artefacts and partial volume averaging artefacts by excluding lesion borders. (**A**) A 45-year-old male, pathologically confirmed as a low-grade (ISUP 1) ccRCC of the right kidney exhibited an ECV of 30.68%; (**B**) A 53-year-old female, pathologically confirmed as a low-grade (ISUP 2) ccRCC of the left kidney exhibited an ECV of 35.34%; (**C**) A 61-year-old male, pathologically confirmed as a high-grade (ISUP 3) ccRCC of the left kidney exhibited an ECV of 41.71%; (**D**) A 73-year-old male, pathologically confirmed as a high-grade (ISUP 4) ccRCC of the left kidney exhibited an ECV of 56.44%. Notes: (**A1**, **B1**, **C1**, **D1**) unenhanced phase images, (**A2**, **B2**, **C2**, **D2**) corticomedullary phase (MP) images, (**A3**, **B3**, **C3**, **D3**) nephrographic phase (NP) images, (**A4**, **B4**, **C4**, **D4**) delayed phase (DP) images, (**A5**, **B5**, **C5**, **D5**) Hematoxylin-Eosin staining magnified images (×200), and (**A6**, **B6**, **C6**, **D6**) Carbonic Anhydrase IX (CAIX) immunohistochemical staining images(×200)

Each radiologist manually delineated a circular ROI on the maximal tumor cross-section, aiming to encompass the largest possible area. The ROI was placed in the most homogeneous and hyperintense portion of the solid tumor, deliberately avoiding regions with necrosis, hemorrhage, calcification, or blood vessels. To ensure data consistency and comparability, the selected lesion was consistently located at the same position across the unenhanced, MP, NP, and DP images (Fig. 2). Furthermore, a circular ROI was positioned within the aorta on the same slice chosen for tumor evaluation. The largest ROIs were placed while ensuring they did not include the vessel wall, particularly avoiding any calcifications or thrombus present. To ensure accuracy, each measurement was repeated three times, and the average value was used for the analysis. Additionally, the mean CT values, measured independently by both radiologists, were calculated and incorporated into the analysis.

The hematocrit (Hct) levels of patients were measured within one week following the CT imaging. The ECV fraction (%) was then computed using the following formula:

ECV (%) = (1 − Hct) × (ΔHU _ccRCC_ / ΔHU _aorta_) × 100%.

Where ΔHU _ccRCC_ and ΔHU _aorta_ represent the absolute enhancement of the ccRCC and aorta during the NP, respectively. These values were obtained by subtracting the CT values in the unenhanced phase from those in the NP.

The measurements for the different phases of contrast-enhanced CT imaging were calculated using the following formulas:

ΔS1 = HU__MP_– HU _unenhanced_.

ΔS2 = HU__NP_– HU _unenhanced_.

ΔS3 = HU__DP_– HU _unenhanced_.

Arterial enhancement fraction (AEF) was calculated using the following equations:

AEF = ΔS1 /ΔS2.

### Histopathological analysis

Resected ccRCC specimens were evaluated by two pathologists with over 10 years of experience, who were blinded to both the clinical data and the results of the CECT imaging. Tumor grading was performed based on the assessment of nucleolar prominence, as outlined in the 2016 WHO/ISUP grading system [17]. The tumors were categorized into two groups: low-grade (ISUP 1–2) and high-grade (ISUP 3–4). In cases of disagreement between the two pathologists, a consensus was reached through discussion.

### Statistical analysis

Statistical analyses were performed using SPSS version 26.0. The Kolmogorov–Smirnov test was used to evaluate the normality of the data distribution. Continuous variables that followed a normal distribution are expressed as mean ± standard deviation, while those with a non-normal distribution are presented as medians along with interquartile ranges. Categorical data are reported as frequencies and percentages (%). The intra-class correlation coefficient (ICC) was calculated to assess the consistency between raters. Differences between the four groups were analyzed with one-way analysis of variance, followed by multiple comparisons using the least significant difference method. Pearson’s correlation was conducted to examine the relationships between variables. Diagnostic performance was evaluated using receiver operating characteristic (ROC) curves, and the areas under the curve (AUC) were compared to determine statistical significance. All statistical tests were two-tailed, with a significance level set at *P* < 0.05.

## Results

### Clinical characteristics and histological results

The histological and clinical characteristics are summarized in Table 1. Histological grading showed that 8 patients had ISUP grade 1 lesions, 34 had grade 2 lesions, 15 had grade 3 lesions, and 8 had grade 4 lesions. The inter-rater reliability of the ECV measurements, evaluated by the two radiologists, was found to be excellent, with an ICC of 0.847 [95% confidence interval: 0.754 to 0.913].

**Table 1:** Clinical characteristics of the participants

Parameters	ISUP grade 1	ISUP grade 2	ISUP grade 3	ISUP grade 4
**Gender (M/F)**	6/2	22/12	9/6	8/0
**Age (years)**	54.50 ± 13.24	57.79 ± 10.32	59.40 ± 11.87	63.00 ± 8.32
**Long diameter(mm)**	40.63 ± 18.56	48.91 ± 21.93	51.53 ± 24.77	76.13 ± 27.37
**Calcification (number)**	3	20	3	4
**Necrotic or cystic (number)**	6	34	14	8
**Nephrectomy (number)**				
**Partial nephrectomy (%)**	6 (9.6%)	19 (29.1%)	8 (12.2%)	0 (0.0%)
**Radical nephrectomy (%)**	2 (3.1%)	15 (23.1%)	7 (10.7%)	8 (12.2%)
**Location (number)**				
**Left (%)**	2 (3.1%)	17 (26.1%)	3 (4.2%)	2 (3.1%)
**Right (%)**	6 (9.6%)	17 (26.1%)	12 (18.2%)	6 (9.6%)
**T stage (number)**				
**T1a**	4	13	4	1
**T1b**	4	16	6	1
**T2a**	0	2	3	2
**T2b**	0	2	0	0
**T3a**	0	0	1	2
**T3b**	0	1	0	2
**T4**	0	0	1	0
**N stage (number)**				
**N1**	1	4	3	2
**M stage (number)**				
**M1**	0	1	2	3
**Hct**	46.39 ± 3.44	43.12 ± 3.94	42.94 ± 3.94	38.73 ± 7.39

Note: Data are presented as the number (**%**) or mean ± standard deviation. ISUP, International Society of Urological Pathologists; Hct, hematocrit

### Clinical and CT parameters between different CcRCC grades

The mean ECV values were 28.31 ± 3.34% for ISUP grade 1, 35.06 ± 4.73% for grade 2, 46.69 ± 4.77% for grade 3, and 56.41 ± 6.36% for grade 4. The differences in ECV across the four ISUP grades were highly significant (*P* < 0.001). Significant differences in CT values were detected across the ISUP grades in the unenhanced scan (*P* = 0.013), NP (*P* < 0.001), DP (*P* = 0.006), ΔS2 (*P* < 0.001), and ΔS3 (*P* = 0.010). No significant differences were observed in the CT values for the MP (*P* = 0.083), ΔS1 (*P* = 0.063), unenhanced phase of the aorta (*P* = 0.995) and balance phase (*P* = 0.127) across the ISUP grades (Table 2).

**Table 2:** Comparison of CT parameter values between four ISUP grades

Parameters	ISUP grade 1	ISUP grade 2	ISUP grade 3	ISUP grade 4	F	*p*
**CT value in plain scan (HU)**	29.17 ± 6.48	33.78 ± 6.80	38.58 ± 6.69^ab^	31.25 ± 8.36^c^	3.893	0.013*
**CT value of MP (HU)**	110.26 ± 49.25	124.70 ± 34.64	147.71 ± 42.15	139.71 ± 15.48	2.330	0.083
**CT value of NP (HU)**	105.49 ± 23.89	125.06 ± 28.72	162.35 ± 21.31^ab^	177.32 ± 13.78^ab^	18.504	< 0.001*
**CT value of DP (HU)**	84.62 ± 18.99	94.76 ± 24.55	112.14 ± 16.07^ab^	112.24 ± 17.05^ab^	4.513	0.006*
**CT value of aorta in plain scan (HU)**	325.85 ± 54.35	323.73 ± 54.35	324.85 ± 44.36	329.08 ± 59.91	0.023	0.995
**CT value of aorta in balance phase (HU)**	171.40 ± 21.28	155.35 ± 29.42	157.52 ± 16.02	141.65 ± 15.08	1.979	0.127
**ΔS1 (HU)**	77.01 ± 49.41	93.19 ± 34.91	116.39 ± 39.95^ab^	108.92 ± 18.51	2.558	0.063
**ΔS2 (HU)**	72.24 ± 24.46	93.55 ± 28.55^a^	131.03 ± 18.39^ab^	146.53 ± 19.37^ab^	19.525	< 0.001*
**ΔS3 (HU)**	55.45 ± 16.31	60.98 ± 21.66	73.57 ± 15.76^ab^	80.99 ± 11.59^ab^	4.107	0.010*
**ECV (%)**	28.31 ± 3.34	35.06 ± 4.73^a^	46.69 ± 4.77^ab^	56.41 ± 6.36^abc^	67.979	< 0.001*
**AEF (%)**	0.98 ± 0.38	1.03 ± 0.38	0.90 ± 0.33	0.75 ± 0.11	1.677	0.181

Notes: Data are presented as the number, mean ± standard deviation. ^a^ significant difference with ISUP grade 1 (*p* < 0.05), ^b^ significant difference with ISUP grade 2 (*p* < 0.05), ^C^ significant difference with ISUP grade 3 (*p* < 0.05). * *p* < 0.05. CT, computed tomography; HU, Hounsfield unit; MP, corticomedullary phase, NP, nephrographic phase; DP, delay phase; ECV, extracellular volume; AEF, arterial enhancement fraction

### Correlation of CT parameters with the pathologic grade of CcRCC

Higher-grade tumors exhibited significantly higher ECV values compared to lower-grade tumors. Spearman correlation analysis showed a positive relationship between ECV values and the pathological grade of ccRCC (*r* = 0.448, *P* = 0.032) (Table 3).

**Table 3:** Correlation analysis between high-grade group and low-grade group of CcRCC and each parameter

Parameters	Low-grade group	High-grade group	*r*	*P*
**ΔS1 (HU)**	98.49 ± 37.99	113.79 ± 33.74	0.203	0.353
**ΔS2 (HU)**	106.09 ± 136.42	136.42 ± 19.79	0.108	0.623
**ΔS3 (HU)**	65.66 ± 20.22	76.15 ± 14.63	-0.143	0.516
**AEF (%)**	0.96 ± 0.35	0.85 ± 0.28	0.219	0.316
**ECV (%)**	39.54 ± 9.81	50.08 ± 7.05	0.448	0.032*

Notes: Data are presented as the mean ± standard deviation. * *p* < 0.05. HU, Hounsfield unit; ECV, extracellular volume; AEF, arterial enhancement fraction

### Screening of CT quantitative parameters

To control for potential confounding factors, tumor length and CT parameters that showed statistical significance in the univariate analysis—namely, the CT values of the MP, NP, and DP phases, as well as ΔS1, ΔS3, and ECV—were incorporated into a multifactorial binary logistic stepwise forward regression analysis. The results of the multifactorial regression indicated that only ECV was a statistically significant predictor (*P* = 0.035), while the other variables were excluded as confounders (Table 4).

**Table 4:** Multifactorial binary logistic Stepwise forward regression analysis of CT quantitative parameters for preoperative prediction of CcRCC stage

CT parameters	Coefficient	SE	Wald	*P*	OR	95%CI
**Long diameter**	-0.016	0.041	0.158	0.691	0.984	0.909 ~ 1.066
**CT value of MP**	-0.055	0.128	0.183	0.668	0.947	0.737 ~ 1.216
**CT value of NP**	0.056	0.038	2.141	0.143	1.057	0.981 ~ 1.139
**CT value of DP**	0.019	0.172	0.012	0.911	1.019	0.728 ~ 1.427
**ΔS1**	0.072	0.131	0.305	0.581	1.075	0.731 ~ 1.390
**ΔS3**	-0.040	0.190	0.045	0.832	0.961	0.662 ~ 1.394
**ECV (%)**	0.669	0.317	4.459	0.035*	1.952	1.049 ~ 3.633

Notes: * *p* < 0.05. CT, computed tomography; H MP, corticomedullary phase, NP, nephrographic phase; DP, delay phase; ECV, extracellular volume. SE standard error; OR odds ratio; CI: confidence interval

### Diagnostic efficiency of CT parameters

**Fig. 3:**
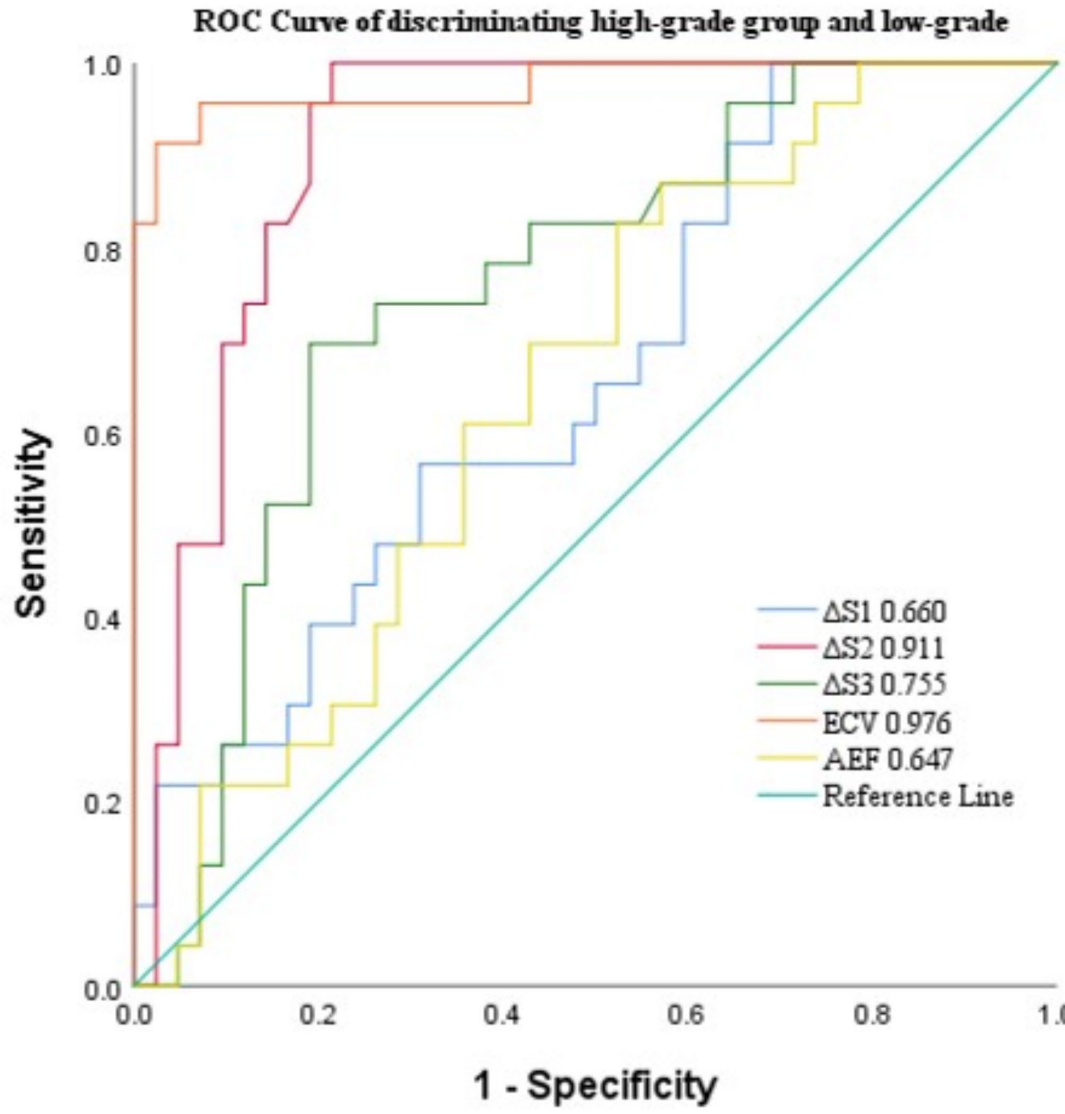
Receiver operating characteristic curves of the diagnostic performance of CT parameters

The ROC curves are shown in Fig. 3. For ECV, the AUC for assessing the low and high pathological grade of ccRCC was 0.976, with a diagnostic threshold set at 41.64. This threshold demonstrated a sensitivity of 91.31% and a specificity of 97.62% (Table 5).

**Table 5:** ROC curve to evaluate the diagnostic efficacy of various parameters in pathological high-grade group and low-grade of CcRCC

Parameters	Cut-off	AUC	*p*	Sensitivity /%	Specificity /%
**ΔS1 (HU)**	64.38	0.660	0.033	100	31.96
**ΔS2 (HU)**	104.45	0.911	< 0.001	100	78.68
**ΔS3 (HU)**	73.79	0.755	0.001	69.57	81.06
**ECV (%)**	41.64	0.976	< 0.001	91.31	97.62
**AEF (%)**	1.12	0.647	0.051	82.61	30.20

Notes: Data are presented as the mean ± standard deviation. HU, Hounsfield unit; ECV, extracellular volume; AEF, arterial enhancement fraction

## Discussion

This study provides evidence that CECT-derived ECV can facilitate noninvasive evaluation and stratification of ccRCC. The findings revealed that ECV values were significantly higher in high-grade ccRCC compared to low-grade tumors. Among all the CT parameters, ECV demonstrated the highest sensitivity, specificity, and diagnostic accuracy in differentiating the pathological grade of ccRCC. Based on these results, we propose that ECV measurement using contrast-enhanced CT is a valuable tool for predicting the pathological grade of ccRCC and for noninvasively distinguishing high-grade from low-grade ccRCC. As an in vivo biomarker, ECV offers additional diagnostic value beyond traditional qualitative CT features.

ccRCC is the most prevalent form of primary renal malignancy, known for its aggressive nature, relatively high rate of metastases, and poorer survival outcomes compared to other subtypes such as papillary and chromophobe RCC [18]. Accurate preoperative prediction of nucleolar grade and stage is crucial for optimizing treatment decisions, assessing the risk of tumor recurrence, and improving prognosis, particularly in avoiding overtreatment of lower grade tumors and minimizing surgical trauma [19]. For low-grade ccRCC patients, minimally invasive approaches, such as radiofrequency ablation or nephron-sparing surgery, are preferred, as they preserve renal function and reduce risks of infection, mortality, and cardiovascular diseases. In contrast, radical interventions are recommended for high-grade ccRCC patients to optimize therapeutic outcomes [1, 2]. Therefore, there is an urgent need for new biomarkers to enable accurate and non-invasive preoperative staging of ccRCC.

The CECT is a standard technique for evaluating renal masses, but its utility in grading ccRCC is limited by the variability and overlap in imaging characteristics. Microvessel density, a key indicator of tumor malignancy, correlates with tumor differentiation, blood supply, and prognosis [20]. High-grade ccRCC is associated with increased microvessel density and blood supply, reflected by higher CT values. However, as the results of this study indicate, the CT values and degree of enhancement observed during the are insufficient to differentiate the pathological grades of ccRCC. The enhancement of HU values is influenced not only by the tumor’s microvascular environment (blood flow, volume, permeability) but also by technical factors (contrast agents, scanning protocols) and patient-specific variations (e.g., body weight, hemodynamics). These factors contribute to the nonspecific nature of conventional enhanced CT findings, complicating the accurate grading of ccRCC. In contrast, ECV provides a more reliable, repeatable, and convenient method for assessing tumor characteristics and grade.

The ECV fraction, a quantitative measure of ECM alterations, reflects changes in the tumor microenvironment. The tumor microvascular environment, which influences malignancy and treatment resistance, is linked to poor outcomes in ccRCC patients [21]. Tumor progression is driven by ECM changes and angiogenesis, which facilitate invasion and metastasis [22, 23]. Cancer-associated fibroblasts, key stromal cells, contribute to ECM stiffening through collagen secretion, promoting tumor spread [24, 25]. ECV provides valuable insights into tumor tissue composition [26]. Elevated ECV correlates with fibrosis and amyloid deposition, enhancing its utility for assessing tissue composition in ccRCC and other cancers. Moreover, increased ECV reflects expanded extracellular space, often associated with abundant microvessels that reduce tumor hypoxia, potentially impairing tumor cell differentiation [27–29]. ECV has been successfully used to evaluate liver and pancreatic fibrosis and tumor angiogenesis, with studies showing strong correlations between imaging-derived ECV and biopsy-determined tumor stroma [30–32]. It is emerging as a promising biomarker for noninvasive tumor assessment, offering stability and independence from imaging parameters, contrast doses, and physiological variations [33]. Previous research has demonstrated a strong correlation between ECV fractions obtained from single-energy and dual-energy CT [31, 34]. The ECV fraction obtained from equilibrium CECT has also been linked to widespread fibrosis in the liver, pancreas, and heart, which is easily integrated into routine clinical practice [35]. Therefore, in this study, we used single-energy equilibrium contrast-enhanced CT to calculate ECV.

In this study, we utilized the ECV fraction calculated from the NP of CECT, which offers the advantage of reducing examination time and radiation exposure. While there is no consensus on the optimal delay time for calculating ECV, the NP phase is a routine contrast-enhanced phase in renal CT protocols and demonstrates significant potential for ECV calculation. Although studies on the use of ECV as a prognostic marker for ccRCC are limited, our findings suggest that CECT-derived ECV is a reliable and easily integrable method for predicting tumor grade. By leveraging standard CECT image acquisition and reconstruction methods, which are commonly accessible, modern multidetector scanners are able to minimize artifacts and noise. This results in improved accuracy for ECV quantification while simultaneously reducing radiation exposure.

Our study found that ECV values were higher in higher-grade tumors, correlating with poorer pathological differentiation and advancing tumor grade. This finding is consistent with the ECV results derived from MRI [8, 14], further supporting the notion that ECV are reliable indicators for differentiating the pathological grades of ccRCC. Thus, ECV is a valuable marker for distinguishing high-grade from low-grade ccRCC. ECV measured in the equilibrium phase shows strong predictive potential and may serve as a reliable quantitative imaging tool for stage ccRCC grading. Higher ISUP grade tumors, especially grade 4 ccRCC, exhibit increased nucleolar polymorphism and sarcomatoid or rhabdoid differentiation [36]. Sarcomatoid dedifferentiation in these tumors is linked to ECM alterations, including abnormal collagen synthesis and assembly, which likely contribute to the observed differences in ECV values between lower- and higher-grade ccRCC [37]. ECV is influenced not only by ECM composition but also by other tissue properties, such as nucleolar characteristics. In higher-grade ccRCC, angiogenesis increases cellularity, micronecrosis, and tumor necrosis, both macroscopic and microscopic, further impacting ECV measurements [38, 39].

This study evaluates the efficacy of CT morphological feature sand ECV models in identifying the pathological grades of ccRCC. The ECV fraction demonstrated significantly higher accuracy than CT morphological features alone in differentiating tumor grades. Combining ECV with morphological features may further enhance grading accuracy, potentially reducing the likelihood of inappropriate treatment decisions. Imaging-based grading could reduce the reliance on renal mass biopsies, thereby supporting treatment selection, immunotherapy planning, and post-resection tumor monitoring. Early intervention for high-grade tumors based on imaging findings could improve patient outcomes and survival rates. Furthermore, this non-invasive approach allows for regular monitoring, enabling timely interventions without the need for repeated biopsies. Future research will focus on exploring the relationship between CT-derived ECV and pathological conditions to further validate its accuracy and reliability.

However, this study has several limitations. First, as a single-center retrospective study with a relatively small sample size, it is subject to potential selection bias. Therefore, future validation in larger, prospective, multi-center cohorts is essential. Second, circular ROIs were used for sampling ccRCC due to software limitations, which may not fully represent the tumor’s heterogeneity, potentially leading to sampling bias. Lastly, pathological data for ECV were not directly obtained to validate the imaging-based measurements. Despite these limitations, standardized scanning parameters and contrast protocols were adhered to, ensuring the comparability of imaging results.

## Conclusion

In conclusion, ECV may serve as a valuable in vivo biomarker for differentiating the grades of ccRCC, providing diagnostic insights beyond conventional qualitative CT features. This finding suggests that ECV derived from CECT can offer crucial information about the tumor microenvironment, potentially aiding in the personalization of treatment for ccRCC. The ability to characterize tumors noninvasively using ECV values holds promise for optimizing individualized treatment strategies, assisting clinicians in selecting less invasive therapeutic options.

## Electronic supplementary material

Below is the link to the electronic supplementary material.

Supplementary Material 1

## Data Availability

No datasets were generated or analysed during the current study.

## Abbreviations

ccRCC: Clear cell renal cell carcinoma
ISUP: International Society of Urological Pathologists
CT: Computed tomography
ECV: Extracellular volume
CECT: Contrast-enhanced CT
ECM: Extracellular matrix
MRI: Magnetic resonance imaging
HU: Hounsfield unit
MP: Corticomedullary phase
NP: Nephrographic phase
DP: Delayed phase
ROI: Region of interest
Hct: Hematocrit
AEF: Arterial enhancement fraction
ICC: Intra-class correlation coefficient
ROC: Receiver operating characteristic
AUC: Areas under the curve
